# A short RNA stem–loop is necessary and sufficient for repression of gene expression during early logarithmic phase in trypanosomes

**DOI:** 10.1093/nar/gku358

**Published:** 2014-05-09

**Authors:** Sandra M. Fernández-Moya, Mark Carrington, Antonio M. Estévez

**Affiliations:** 1 Instituto de Parasitología y Biomedicina ‘López-Neyra’, IPBLN-CSIC, Parque Tecnológico de Ciencias de la Salud, Avda. del Conocimiento, s/n, 18016 Armilla, Granada, Spain; 2 Department of Biochemistry, University of Cambridge, Tennis Court Road, Cambridge CB2 1QW, UK

## Abstract

We have compared the transcriptomes of cultured procyclic *Trypanosoma brucei* cells in early and late logarithmic phases and found that ∼200 mRNAs were differentially regulated. In late log phase cells, the most upregulated mRNA encoded the nucleobase transporter NT8. The 3′ untranslated region (UTR) of *NT8* contains a short stem–loop *cis*-element that is necessary for the regulation of *NT8* expression in response to external purine levels. When placed in the 3′-UTR of an unregulated transcript, the *cis*-element is sufficient to confer regulation in response to purines. To our knowledge, this is the first example of a discrete RNA element that can autonomously regulate gene expression in trypanosomes in response to an external factor and reveals an unprecedented purine-dependent signaling pathway that controls gene expression in eukaryotes.

## INTRODUCTION

Coordinating expression of functionally-related genes in response to environmental signals is essential for a cell. Recent genome-wide studies have shown that the global regulation of gene expression depends largely on post-transcriptional events (1). Key players in post-transcriptional regulation are *trans*-acting factors such as ribonucleic acid (RNA)-binding proteins, non-coding RNAs and metabolites and *cis*-acting elements, which are regulatory sequences usually found within the untranslated regions (UTRs) of messenger RNAs (mRNAs). The interaction between *tran*s- and *cis*-acting factors results in the coordinated regulation of subsets of mRNAs encoding functionally-related proteins, which are known as post-transcriptional operons or regulons (1).

Post-transcriptional regulation is of unusual importance in trypanosomatids. The best characterized members of this group of protozoans are parasites that cause severe diseases in humans and cattle, such as *Trypanosoma brucei*, *Trypanosoma cruzi* and *Leishmania* ssp. (2). They exhibit complex life cycles alternating between insects and mammals, and therefore regulate their gene expression programs in order to adapt to the changes in temperature, pH, nutrients and defenses they find within one or the other host (3). Evidence so far indicates that there is no selective regulation of transcription of protein coding genes by RNA polymerase II. Instead, trypanosomatids rely mainly on mRNA processing, turnover, translation and localization to remodel the proteome during the life cycle (4–6). This is thought to be achieved through the orchestrated interaction of RNA-binding proteins and *cis*-acting RNA elements, and indeed several lines of evidence strongly support a model in which RNA operons operate in these organisms (4,7). In spite of the dependence on post-transcriptional mechanisms, very few RNA-binding proteins have been characterized in detail in trypanosomatids, and in even fewer instances precisely defined *cis*-acting elements have been unequivocally identified as being necessary and sufficient to confer regulation (5,8). These include a 26-mer stem–loop within *T. brucei EP1* procyclin that causes RNA instability in the bloodstream form (9), a 25-nt glycerol-responsive element in the *T. brucei GPEET* procyclin that controls expression by glucose and during development in the insect vector (10), a 50-nt AU-rich region within *mucin* genes in *T. cruzi* that affects mRNA degradation and translation (11) and a 34-nt element that represses expression in proliferative *T. brucei* slender bloodstream forms and activates it in quiescent stumpy bloodstream parasites (12). These *cis*-elements have been shown to be necessary but none has been shown to be functionally sufficient when placed in a different mRNA context. In *Leishmania major*, a retroposon-derived sequence of ∼600 nt located within the 3′-UTR of many transcripts, named SIDER2, has been shown to promote mRNA instability when placed downstream of a reporter gene (13,14). A 79-nt signature was shown to be essential for mRNA degradation (15), but whether these 79 nt are sufficient independently is not known at present. In *Leishmania mexicana*, a 10-nt element termed paraflagellar rod (PFR) regulatory element (PRE) was found to destabilize a *PFR* chimeric transcript containing a heterologous 3-UTR transcribed from an episome (16). However, since sequences corresponding to both the 5′-UTR and the open-reading frame of the *PFR* gene were still present in the episomal expression construct, it is still unknown whether the PRE could promote regulation when placed in a different genetic context. Lastly, an octamer sequence was identified in *Crithidia fasciculata* that could confer periodic accumulation on a heterologous transcript, but only when multiple copies of the element were inserted within the 5′-UTR of a reporter gene (17).

In a search to identify novel *cis*- and *trans*-acting factors that regulate gene expression in *T. brucei*, we compared the transcriptome of procyclic *T. brucei* cells in early and late logarithmic phases using high-throughput sequencing (RNAseq). The most upregulated transcript in late log phase encodes the purine transporter NT8. We have identified a short RNA element within the 3′-UTR of *NT8* that is necessary for purine-dependent repression of expression in early log phase, and found that it is also sufficient to confer regulated expression when placed in a different genetic context.

## MATERIALS AND METHODS

### Trypanosome culture

*T. brucei* Lister 427 procyclic cells were grown at 27ºC in SDM-79 medium (18) containing 10% fetal bovine serum. Purine-deprived SDM-79 was supplemented with 10% dialyzed fetal bovine serum (Invitrogen). When used, guanosine was added at a concentration of 100 μM. Transgenic trypanosomes were obtained following standard procedures (19).

### RNA and transcriptome analysis

Total RNA was extracted using peqGOLD Trifast (peqlab). Northern hybridizations, mRNA degradation experiments and quantitative reverse transcriptase-polymerase chain reaction (RT-PCR) were done as previously described (20,21). Complementary deoxyribonucleic acid libraries for high-throughput sequencing were obtained from two biological replicates using the Illumina mRNA-seq kit according to manufacturer's instructions. Libraries were sequenced on an Illumina platform at EASIH (Eastern Sequencing and Informatics Hub, Cambridge, UK) and the reads of 36 nucleotides in length were aligned to the *T. brucei* TREU 927 genome v4 using Bowtie (22,23). Manipulation and processing of the aligned tags were done using SAMtools (24). Aligned sequence tags were visualized using Artemis (25). The number of hits per open reading frame (ORF) was determined using HT-Seq software (http://www-huber.embl.de/users/anders/HTSeq/doc/index.html). The number of reads mapping to each feature in each condition was compared to early log samples using the EdgeR package (26). Gene Ontology (GO) assignments were done using GOStat (27). RNA secondary structure predictions were obtained using RNAfold (http://rna.tbi.univie.ac.at/cgi-bin/RNAfold.cgi).

### Luciferase constructs and assays

Luciferase expression constructs were derived from pGR108 (20) (Supplementary Figure S1A). To obtain the *NT8* 3′-UTR, a fragment spanning the complete intergenic region between genes Tb917.11.3620 and Tb917.11.3630 was amplified by PCR (Supplementary Figure S1A). Intact and deleted versions of the *NT8* 3′UTR were cloned as *Bam*H I-*Hpa* I fragments into pGR108 digested in the same way. *NT8* 5′UTR was cloned as a blunt-*Hin*d III fragment into pGR108 digested with *Sma* I and *Hin*d III. Plasmids were digested with *Not* I and stably integrated in the tubulin locus by electroporation as described (20). A list of the plasmids used in this work is shown in Supplementary Figure S1B).

For luciferase assays, 2–4×10^7^ cells were washed in phosphate-buffered saline and frozen at −80ºC until use. Cell pellets were thawed, resuspended in lysis buffer (100-mM potassium phosphate, pH 7.8, 2 mM-dithiothreitol (DTT), 0.1% Triton X-100, 10% glycerol) at a concentration of 0.1 ml per 1×10^7^ cells, vortexed briefly, incubated 5 min on ice and centrifuged at 16 000*g* for 10 min at 4ºC. Ten to twenty microliters were used per assay using Promega's Luciferase Assay System in a Berthold FB 12 luminometer according to manufacturer's instructions. Protein concentration was determined using Bradford reagent (BioRad).

## RESULTS

### Transcriptome analysis of procyclic trypanosomes in early and late logarithmic phases

**Figure 1.**
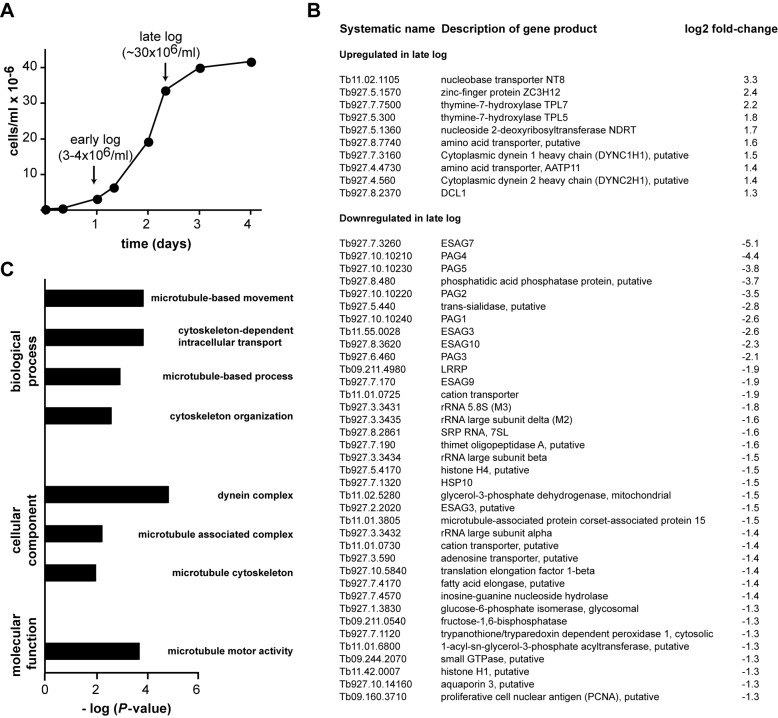
The transcriptome of procyclic *T. brucei* is remodeled during the growth curve. (**A**) Typical growth curve of procyclic *T. brucei* cells. RNA samples were obtained from cells in early and late logarithmic phases, and subjected to high-throughput sequencing. (**B**) List of mRNAs whose abundance is altered at least ± 2.5-fold (log_2_ = ±1.3) in late log phase. Transcripts encoding hypothetical proteins are not shown. A complete list can be found in Supplementary Table S1. (**C**) Gene Ontology (GO) analysis of mRNAs upregulated in late log phase. Only significantly enriched categories (*P* < 0.01) are shown.

To analyze how the transcriptome of procyclic trypanosomes is remodeled during the growth cycle in culture, we prepared RNA samples from early and late logarithmic phase cells (Figure 1A) and compared them using deep sequencing. There were ∼200 transcripts with at least 2-fold altered abundance in late logarithmic phase, of which 80 were upregulated and 117 downregulated. A list of the top (>2.5-fold) regulated transcripts with known or predicted functions is shown in Figure 1B. The complete list of all regulated transcripts, including hypothetical proteins, can be found in Supplementary Table S1.

GO was used to categorize regulated transcripts according to their biological process, cellular component and molecular function. Significantly enriched GO terms (*P* < 0.01) for upregulated transcripts are shown in Figure 1C. There was a significant enrichment for transcripts encoding motor proteins such as microtubule components and dynein complexes. On the other hand, there was no significant enrichment for any GO category in the downregulated mRNA set. However, many RNA-polymerase I transcripts (i.e. procyclic-associated genes and ribosomal RNAs) decreased in abundance in late logarithmic phase, a phenomenon also described in yeast cells entering stationary phase (28,29).

### mRNA half-life is regulated during the growth cycle

**Figure 2.**
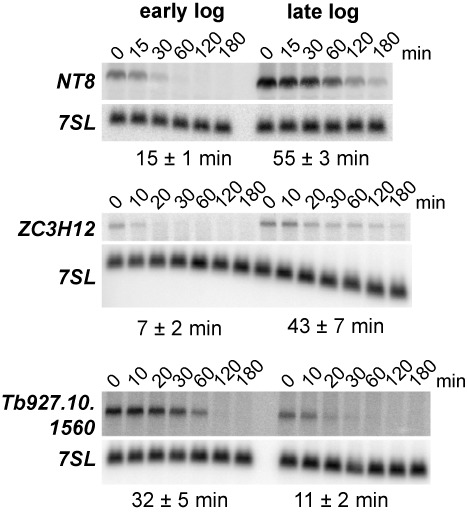
mRNA turnover is regulated during the growth curve. Half-lives of two transcripts upregulated in late log phase (*NT8* and *Z3CH12*) and one downregulated in late log phase (*Tb927.10.1560*) were measured as previously described (20) from data obtained from at least three independent experiments. Representative northern blots are shown in each case. The signal recognition particle *7SL* RNA was used for normalization.

To assess whether the observed changes in mRNA abundance were due to alterations in RNA degradation rates, we measured the half-life of three transcripts, two upregulated in late log phase, *NT8* (Tb11.02.1105) and *ZC3H12* (Tb927.5.1570) (Figure 1B), and one transcript downregulated in late log phase, *Tb927.10.1560* (Supplementary Table S1). As shown in Figure 2, the half-lives of both upregulated transcripts increased, whereas it decreased in the case of the downregulated mRNA. These results indicate that the changes in mRNA steady-state levels result from different half-lives in early and late log phase cells.

### External purine concentration regulates mRNA abundance

**Figure 3.**
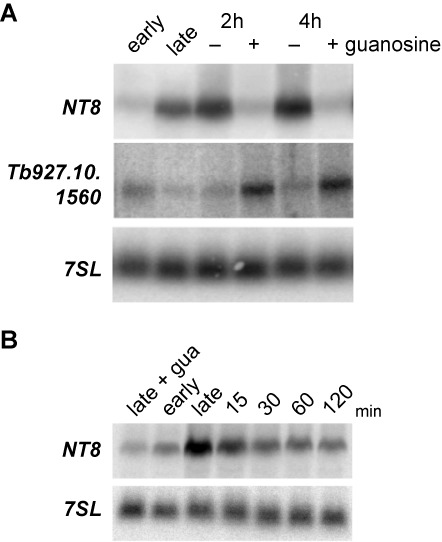
Extracellular purine levels regulate mRNA abundance. (**A**) A culture in late log phase was split in two subcultures, and 100-μM guanosine was added to one of them. RNA samples were obtained after 2-h or 4-h incubation at 27ºC, and the abundance of *NT8* and *Tb927.10.1560* was analyzed by northern blot using specific probes. The signal recognition particle *7SL* RNA was used for normalization. (**B**) *NT8* levels were also determined at shorter incubation times after the addition of guanosine. The addition of 100-μM guanosine to early log cultures prevented the accumulation of *NT8* later on in late log phase (leftmost lane).

**Figure 4.**
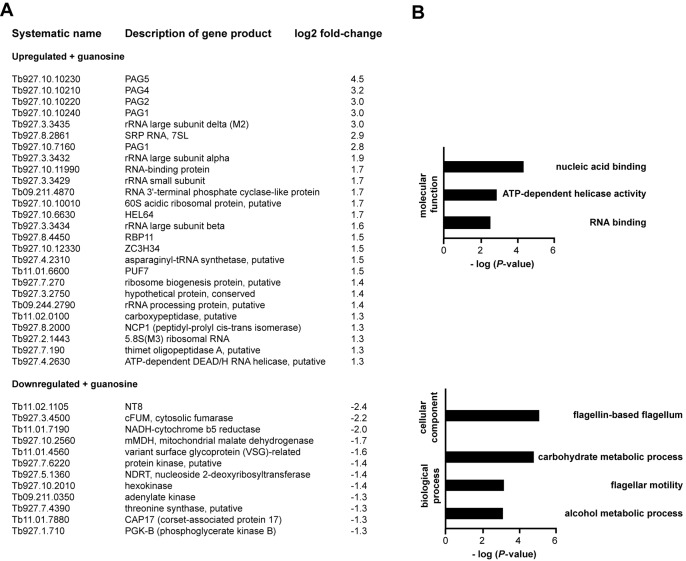
Effect of guanosine addition on the transcriptome of late log procyclic trypanosomes. **(A)** A list of mRNAs whose abundance is altered at least ± 2.5-fold (log_2_ = ±1.3) upon addition of guanosine to late log cells. Transcripts encoding hypothetical proteins are not shown. A complete list can be found in Supplementary Table S2. **(B)** Gene Ontology (GO) analysis of regulated mRNAs. Only significantly enriched categories (*P* < 0.01) are shown.

The most upregulated mRNA in late log phase was *NT8* (Figure 1B) that encodes a permease that transports different purines with high affinity (30,31). We wondered whether the addition of purines in late log phase could reverse the increased abundance of *NT8* mRNA. Indeed, when guanosine was added to late log cultures, the abundance of *NT8* decreased to levels close to those observed in early log phase (Figure 3A): *NT8* levels increased 9.1 ± 1.7-fold (*n* = 3) when entering in late log phase and decreased back 8.5 ± 1.3-fold upon addition of the nucleoside. A similar effect was observed when another purine, hypoxanthine, but not a pyrimidine, thymidine, was used (data not shown). Moreover, the mRNA *Tb927.10.1560*, which is downregulated 1.8 ± 0.5-fold in late log phase, increased in abundance 2.4 ± 0.4-fold when purines were added to the culture (Figure 3A). Remarkably, the response to guanosine was very fast. *NT8* mRNA levels were reduced 2.4 ± 0.6-fold 15 min after addition of guanosine to the medium (Figure 3B). The addition of additional guanosine to early log cultures prevented the accumulation of *NT8* later on in late log phase (Figure 3B, leftmost lane). NT8 was apparently the only purine transporter regulated during the growth cycle (Supplementary Table S1). We checked the expression of a different purine transporter, NT10 (32), and found that its expression was not altered during the growth curve (data not shown). The effect of purine addition to late log cultures was analyzed in more detail using RNAseq transcriptome analysis. There were 138 transcripts with at least 2-fold altered abundance in guanosine-supplemented medium, of which 95 were upregulated and 43 downregulated. A list of the most regulated (>2.5-fold) transcripts with known or predicted functions is shown in Figure 4A. The complete list of regulated transcripts, including hypothetical proteins, can be found in Supplementary Table S2. GO analysis (Figure 4B) showed that RNA-binding proteins, especially helicases, were significantly upregulated in guanosine-supplemented medium. RNA-polymerase I-derived transcripts also increased in abundance in the presence of guanosine. On the other hand, transcripts encoding enzymes involved in carbohydrate metabolism and flagellar motility were significantly enriched in the downregulated set.

### The 3′-UTR of *NT8* confers regulation during the growth cycle

**Figure 5.**
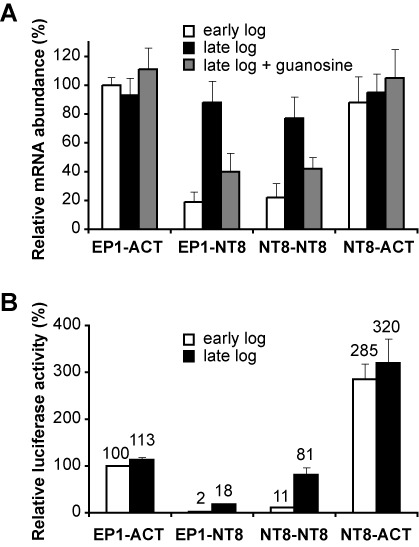
The 3′-UTR of *NT8* confers regulation to a luciferase reporter gene. Stable cell lines were generated that expressed luciferase under the control of *NT8* 5′- and/or 3′-UTRs. A cell line expressing luciferase fused to procyclin *EP1* 5′-UTR and actin 3′-UTR was used as a control. Luciferase mRNA (**A**) or activity (**B**) levels were normalized to those of the control cell line in early log phase, which were set at 100%. Transcript levels were determined by quantitative RT-PCR. The actual values of relative luciferase activity are indicated above each bar for convenience.

Most regulatory RNA elements affecting mRNA half-life and translation have been detected in the 3′-UTR in trypanosomatid genes (5). To identify *cis*-acting elements responsible for the observed regulation along the growth curve, we tested the 5′-UTR and/or the 3′-UTR of *NT8* mRNA into a luciferase reporter transgene that was integrated to form stable cell lines. As a control, we used a cell line transfected with plasmid pGR108, which expressed luciferase under the control of the *EP1* procyclin 5′-UTR and the *actin* 3′-UTR [(20), Supplementary Figure S1]. In this cell line, neither luciferase mRNA nor activity was differentially regulated during the growth curve (Figure 5). In all experiments described in this work, luciferase mRNA and activity levels in reporter transgenes were normalized to those of actin 3′-UTR in early log phase set at 100%. When the *NT8* 3′-UTR was fused to luciferase, a 5-fold decrease in transcript levels was observed in early log phase as compared with the control cell line. Moreover, luciferase mRNA levels increased 4-fold during late log phase and decreased again 2 h upon addition of guanosine to the culture medium (Figure 5A). Replacement of the *EP1* 5′-UTR with the *NT8* 5′-UTR did not have any apparent effect on mRNA abundance (Figure 5A). At the protein level, the presence of *NT8* 3′-UTR caused an ∼50-fold reduction of luciferase activity during early log phase; the activity increased 9-fold upon entering in late log phase (Figure 5B). When luciferase was expressed under the control of both the 5′- and 3′-UTRs of *NT8*, protein levels were 4- to 5-fold higher than those detected when using the 3′-UTR alone in both growth phases (Figure 5B). This suggested that the 5′-UTR of *NT8* enhances translation. Indeed, when luciferase was expressed under the control of the *NT8* 5′-UTR alone, an ∼3-fold increase in luciferase activity was observed (Figure 5B), suggesting that the 5′-UTR of *NT8* contains RNA elements that stimulate translation regardless of the growth phase. The results also show that the 3′-UTR of *NT8* contains regulatory RNA elements that confer both repression of gene expression during early log phase and responsiveness to purine levels in the medium. With this experimental design, we could not assess the effect of purines on luciferase activity, since there was no enough time for the protein to be turned over after the addition of guanosine (data not shown). This was probably due to the stability of luciferase in procyclic cells (33). Nevertheless, luciferase activity could be indeed modulated by purine levels (see below).

### Identification of regulatory elements within the *NT8* 3′-UTR

**Figure 6.**
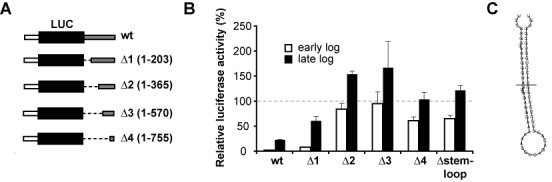
Identification of RNA regulatory elements within the 3′-UTR of *NT8*. (**A**) Serial deletions of the *NT8* 3′-UTR were generated starting from the 5′ end and fused to a luciferase gene with an *EP1* 5′-UTR. (**B**) Luciferase activity was normalized to that of a cell line expressing the reporter under the control of *EP1* 5′-UTR and *actin* 3-UTR (dashed line). (**C**) Predicted secondary structure of the regulatory stem–loop identified in deletion Δ2. RNAfold plots corresponding to the entire 3′-UTR can be seen in Supplementary Figure S2.

To identify RNA regulatory sequences in the 3′-UTR of *NT8*, we performed serial deletions of the 3′-UTR and fused them to a luciferase reporter gene (Figure 6A). Deletion of the first 203 nt did not have any apparent effect on luciferase activity (deletion Δ1, Figure 6B). However, when the first 365 nt were removed, repression during early log phase was lost (deletion Δ2). Further deletions in the 3′-UTR (deletions Δ3 and Δ4) did not have additional effects on expression in comparison with those observed in deletion Δ2 (Figure 6B). These results suggested that there is a regulatory element located between nucleotides 204 and 365 in the 3′-UTR of *NT8*. Secondary RNA structure predictions using RNAfold (Supplementary Figure S2A) revealed a 60-nt stem–loop structure with very high base-pairing probabilities, which is shown in detail in Figure 6C. Removal of just these 60 nt did not apparently alter the secondary structure of the 3′-UTR (Supplementary Figure S2B) and had virtually the same effect as deletions Δ2 to Δ4 (Figure 6B). This suggests that the short stem–loop structure is responsible for most of the observed repression of gene expression during early log phase. There are three genes encoding NT8 arranged in a tandem array in the *T. brucei* genome (Supplementary Figure S1), and all are predicted to contain the stem–loop within their 3′-UTRs (data not shown).

### The stem–loop element represses gene expression in early log phase and confers purine-dependent regulation

**Figure 7.**
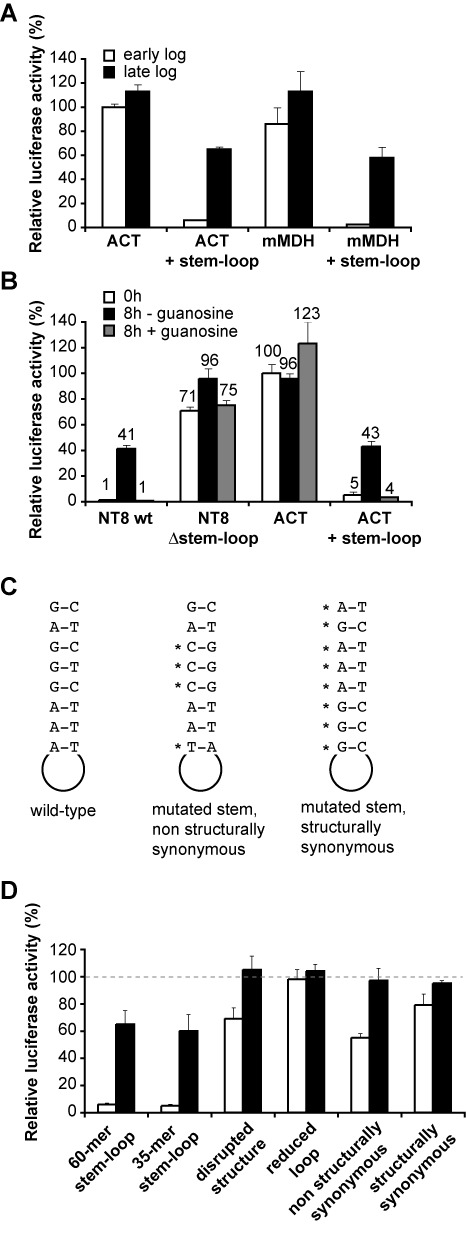
Effect of the stem–loop element on gene expression in different gene contexts. (**A**) Cell lines were generated which expressed luciferase under the control of actin (*ACT*) or mitochondrial malate dehydrogenase (*mMDH*) 3′-UTRs in which the regulatory stem–loop was inserted. (**B**) Effect of guanosine withdrawal in early log cultures expressing luciferase under the control of 3′-UTRs containing or lacking the regulatory stem–loop. The actual values of relative luciferase activity are indicated above each bar for convenience. (**C**) Sequences of the short (35 mer) wild-type stem–loop and mutated versions thereof. Mutations in the stem are indicated by asterisks. Detailed sequences and structures can be seen in Supplementary Figures S3 and S4. (**D**) Effect of shorter and mutant stem–loop versions on luciferase expression. Luciferase activity was normalized to that of a cell line expressing the reporter under the control of *EP1* 5′-UTR and *actin* 3-UTR (dashed line).

To study whether the RNA element was sufficient to provide regulation in a different gene context, we modified the luciferase transgene construct to insert the stem–loop 149 nucleotides downstream the luciferase stop codon. As shown in Figure 7A, luciferase activity decreased ∼10-fold in early log phase when the regulatory element was placed into the wild-type *actin* 3′-UTR. In a second experiment, the stem–loop was inserted within a 3′-UTR derived from the mitochondrial malate dehydrogenase (*mMDH*) gene, which was not normally regulated during growth cycle (Supplementary Table S1). Cells expressing luciferase fused to wild-type *mMDH* 3′-UTR showed an activity close to that observed in the actin control cell line (Figure 7A). However, the insertion of the stem–loop within the 3′-UTR of *mMDH* resulted in a 20-fold decrease in luciferase activity in early log phase.

We next studied whether the stem–loop could also confer regulation in response to extracellular purine levels. We used cell lines expressing transgenes encoding luciferase fused to: (i) wild-type *NT8* 3′-UTR, or (ii) *NT8* 3′-UTR without the stem–loop, or (iii) wild-type *actin* 3′-UTR, or (iv) *actin* 3′-UTR containing the stem–loop (Figure 7B). Cells were harvested in early log phase (time 0 h), washed and resuspended in purine-depleted medium. Cultures were then split into two, guanosine was added to one but not the other and luciferase activity was measured after an incubation of 8 h. As shown in Figure 7B, luciferase expression was strongly repressed in a cell line expressing the reporter under the control of an intact *NT8* 3′-UTR when guanosine was present in the medium, and increased ∼40-fold when guanosine was withdrawn. However, when the stem–loop element was absent, no regulation could be observed regardless the presence or absence of guanosine. Moreover, insertion of the stem–loop within the 3′-UTR of the control *actin* gene rendered it responsive to guanosine, since luciferase expression was again inhibited in the presence of guanosine and enhanced in depleted medium. All these results show that the stem–loop element is both necessary and sufficient to promote repression of gene expression during early log phase in response to purine levels.

We also tested whether a shorter version of the stem–loop was still able to repress luciferase activity and found that a hairpin as short as 35 nt (containing the sequence below the horizontal line in Figure 6C) had a similar effect on luciferase expression in comparison with its 60-mer counterpart (Figure 7C). In addition, we generated different mutant versions of the 35-mer stem–loop and analyzed whether they could still mediate repression in early log phase (detailed sequences and predicted structures are available in Supplementary Figures S3 and S4). A point mutation within the loop predicted to disrupt the whole stem–loop structure (Supplementary Figure S3) resulted in a loss of regulation (Figure 7D). We also reduced the loop length to 3 nt and introduced structurally non-synonymous or synonymous mutations in the stem. Regulation was lost in all cases (Figure 7D), suggesting that probably both sequence and structural features are important.

## DISCUSSION

We have used high-throughput sequencing to analyze how trypanosomes remodel their transcriptome when entering in late logarithmic phase, and thus obtain valuable information that could be used to identify novel *cis*- and *trans*-acting factors involved in post-transcriptional regulation. We found that the abundance of ∼200 transcripts was altered during the growth cycle. A previous study using microarrays compared the transcriptome of trypanosomes in logarithmic phase with that of non-dividing cells in stationary phase (34). These authors identified a set of ∼900 regulated mRNAs with no obvious functional relationship. The larger number of regulated genes in their study could be explained if one takes into account the general decline in all cell growth functions that occurs in stationary phase.

The most upregulated mRNA in late log phase encoded the high-affinity purine transporter NT8, not previously identified as upregulated (34). Since trypanosomes are unable to synthesize purines *de novo* (35), it is conceivable that they need to stimulate the transport of nucleobases when purines become scarce as cell density increases. Indeed, it has been shown that trypanosomes and *Leishmania* respond to purine starvation by increasing their transport capacity (36,37). Interestingly, we found that the addition of guanosine to late log cultures reversed the accumulation of *NT8* mRNA and increased the abundance of an otherwise downregulated mRNA. Moreover, we showed that ∼140 transcripts were altered in abundance in guanosine-supplemented medium. Thus, purines act as an extracellular cue that controls gene expression in trypanosomes, as it has been recently reported in *Leishmania* (38).

We identified a short RNA element within *NT8* that represses the expression of this transcript during early log phase. It contains a predicted stem structure consisting of a stretch of purines paired with pyrimidines in the opposite strand and a loop sequence (Figure 6C). Similar purine-pyrimidine double-stranded structures followed by regions of unpaired nucleotides could also be detected in the 3′-UTRs of other regulated transcripts, although there seems to be no conservation at the sequence level (Supplementary Figure S5). In contrast to other *cis*-acting motifs described so far in trypanosomatids, the stem–loop described in this work is sufficient to confer regulated gene expression when placed in different contexts, that is, within the 3′-UTR of transcripts that are not regulated during the growth cycle.

The RNA element identified in this work is able to provide regulation in a purine-dependent manner. In two species of the related trypanosomatid *Leishmania*, purine starvation triggers the transition toward metacyclic forms, the stage responsible for the initiation of infection in the vertebrate host (39). It is therefore conceivable that the same scenario applies to *T. brucei* and purine depletion triggers differentiation to mesocyclic or other developmental stages (40,41). In addition, *in vitro* cultures of trypanosomes in late log phase could be mimicking the environment faced by procyclic cells in the midgut of the insect vector. Thus, the results presented here could be useful to understand how trypanosomes regulate gene expression in order to survive in the different environments.

How does the RNA element function? There are two possible mechanisms: either direct binding of nucleotide or via*trans*-acting factors. Bacterial riboswitches are RNA regions that regulate gene expression by adopting different conformations upon binding to specific metabolites (42). There is no resemblance of the RNA element described here to bacterial purine riboswitches, which are longer in sequence and more complex in structure (43). However, a 34-mer bacterial riboswitch consisting only of a stem–loop structure and a short tail has been described which selectively binds to the nucleoside queuosine (44). Alternatively, specific RNA-binding proteins could bind to the regulatory RNA element in a purine-dependent manner in trypanosomes. For example, a signaling cascade could modify an RNA-binding protein via phosphorylation, methylation or other post-translational modification, which in turn would alter the binding capacity of the protein depending on purine levels. The actual mechanism remains to be determined. Our work has uncovered an unprecedented mechanism by which extracellular purines are able to control gene expression at the RNA level in eukaryotes, and provides an interesting venue for the discovery of novel regulatory molecules and mechanisms in the post-transcriptional world.

## SUPPLEMENTARY DATA

Supplementary Data are available at NAR Online.

SUPPLEMENTARY DATA
