# Supplementary material for: A short RNA stem–loop is necessary and sufficient for repression of gene expression during early logarithmic phase in trypanosomes
Source: Nucleic Acids Res. 2014 May 9;42(11):7201–9. doi: 10.1093/nar/gku358 (PMC4066783; doi:10.1093/nar/gku358)
Supplement: SUPPLEMENTARY DATA [file supp_42_11_7201__index.html]

SUPPLEMENTARY DATA 

# A short RNA stem–loop is necessary and sufficient for repression of gene expression during early logarithmic phase in trypanosomes

## SUPPLEMENTARY DATA

**Files in this Data Supplement:**

- Supplementary Data
