# Supplementary material for: A short RNA stem–loop is necessary and sufficient for repression of gene expression during early logarithmic phase in trypanosomes
Source: Nucleic Acids Res. 2014 May 9;42(11):7201–9. doi: 10.1093/nar/gku358 (PMC4066783; doi:10.1093/nar/gku358)
Supplement: SUPPLEMENTARY DATA [file supp_gku358_nar-00078-f-2014-File009.pdf]

Supplementary Table S1. List of transcripts regulated during the growth cycle.
Transcripts whose abundance is altered at least ± 2-fold (log2 fold change = ±1.0) in late logarithmic phase are shown.

Upregulated in late log

Systematic name	Description of gene product	log2 FC
Tb11.02.1105	NT8.1	3.3
Tb927.5.1570	ZC3H12	2.4
Tb927.7.7500	TPL7, thymine-7-hydroxylase, putative	2.2
Tb927.5.300	TPL5, thymine-7-hydroxylase, putative	1.8
Tb927.5.1360	NDRT, nucleoside 2-deoxyribosyltransferase	1.7
Tb09.160.0900	hypothetical protein, conserved	1.7
Tb927.2.5530	hypothetical protein, conserved, P-loop	1.6
Tb927.5.4020	hypothetical protein	1.6
Tb927.8.7740	amino acid transporter, putative	1.6
Tb927.2.5610	hypothetical protein, conserved, P-loop	1.6
Tb927.8.2060	hypothetical protein, conserved, BEIGE/BEACH	1.5
Tb927.5.1780	hypothetical protein, conserved	1.5
Tb927.7.3160	Cytoplasmic dynein 1 heavy chain (DYNC1H1), putative	1.5
Tb11.18.0001	hypothetical protein, conserved	1.4
Tb927.5.1120	hypothetical protein, conserved	1.4
Tb927.10.13530	hypothetical protein	1.4
Tb927.4.4730	AATP11	1.4
Tb927.4.560	Cytoplasmic dynein 2 heavy chain (DYNC2H1), putative	1.4
Tb927.2.5540	hypothetical protein, conservedm Divergent kinesin	1.4
Tb927.3.4610	hypothetical protein, conserved	1.4
Tb927.8.5580	hypothetical protein, conserved, vacuolar sorting	1.3
Tb927.8.5850	hypothetical protein, conserved	1.3
Tb927.8.7990	hypothetical protein, conserved, Zn-finger & ubiquitin-like	1.3
Tb927.8.2370	DCL1	1.3
Tb927.4.310	hypothetical protein, conserved	1.3
Tb11.01.2430	hypothetical protein, conserved	1.3
Tb11.52.0002	hypothetical protein, conserved	1.3
Tb11.02.0880	hypothetical protein, conserved	1.2
Tb927.8.4780	hypothetical protein, conserved	1.2
Tb11.52.0018	phospholipid-transporting ATPase, putative	1.2
Tb927.10.13330	PFC20, paraflagellar rod component	1.2
Tb11.02.3740	receptor-type adenylate cyclase GRESAG 4, putative	1.2
Tb927.10.15730	hypothetical protein, conserved, ankyrin repeat motif	1.2
Tb927.10.15970	triacylglycerol lipase, putative	1.2
Tb09.211.4760	metacaspase 5, putative	1.2
Tb11.01.7620	hypothetical protein, conserved	1.2
Tb927.10.10280	microtubule-associated protein, putative	1.2
Tb927.6.200	receptor-type adenylate cyclase GRESAG 4, putative	1.1
Tb927.8.1360	hypothetical protein, conserved, dyneyin motif and P-loo	1.1

Tb927.10.5880	Proteophosphoglycan, putative	1.1
Tb927.2.3660	PFC10, paraflagellar rod component	1.1
Tb927.5.2330	hypothetical protein, conserved, armadillo-type fold	1.1
Tb927.2.5270	dynein heavy chain, putative	1.1
Tb927.6.4490	hypothetical protein, conserved	1.1
Tb927.3.4500	cFUM, cytosolic fumarase	1.1
Tb927.7.7200	hypothetical protein, conserved	1.1
Tb927.10.9040	hypothetical protein, conserved	1.1
Tb927.4.620	hypothetical protein, conserved	1.1
Tb927.8.830	hypothetical protein, conserved, EP DOMAIN	1.1
Tb11.02.2090	hypothetical protein, conserved, armadillo-type fold	1.0
Tb927.2.2650	hypothetical protein, conserved	1.0
Tb927.8.6360	hypothetical protein, conserved, SPla/RYanodine receptor	1.0
Tb927.6.4890	METK1 (MAT2), S-adenosylmethionine synthetase	1.0
Tb927.10.1120	hypothetical protein, conserved	1.0
Tb927.3.1310	hypothetical protein, conserved	1.0
Tb09.211.4440	hypothetical protein, conserved	1.0
Tb927.5.3780	hypothetical protein, conserved	1.0
Tb11.01.0390	dynein heavy chain	1.0
Tb927.10.10050	hypothetical protein, conserved	1.0
Tb927.6.620	hypothetical protein, conserved, CH-domain motif	1.0
Tb927.10.15750	hypothetical protein, conserved zinc finger, RING motif	1.0
Tb11.01.5930	hypothetical protein, conserved, Zinc finger, ZZ-type motif	1.0
Tb927.7.310	hypothetical protein, conserved, thioredoxin-fold	1.0
Tb927.10.7890	hypothetical protein, conserved	1.0
Tb927.8.7260	kinetoplast-associated protein, putative	1.0
Tb927.10.8390	hypothetical protein, conserved, HECT domain	1.0
Tb09.160.1400	hypothetical protein, conserved	1.0
Tb927.1.3890	hypothetical protein	1.0
Tb927.10.840	hypothetical protein, conserved, WD40 motif	1.0
Tb927.5.3330	hypothetical protein, conserved	1.0
Tb06.3A7.270	hypothetical protein, conserved	1.0
Tb927.10.8050	TFIIF-stimulated CTD phosphatase	1.0
Tb927.3.1420	hypothetical protein, conserved	1.0
Tb927.6.5150	hypothetical protein, conserved	1.0
Tb927.10.4170	hypothetical protein, conserved, Type III secretion sytem	1.0
Tb927.10.15400	kinesin, putative	1.0
Tb927.5.3160	protein kinase, putative	1.0
Tb927.8.5390	CRK4, cell division control protein 2 homolog 4	1.0
Tb927.8.3250	dynein heavy chain, putative	1.0
Tb927.8.2430	hypothetical protein, conserved	1.0

Downregulated in late log

Systematic name	Description of gene product	log2 FC
Tb927.7.3260	ESAG7	-5.1
Tb927.10.10210	PAG4	-4.4
Tb927.10.10230	PAG5	-3.8
Tb927.8.480	phosphatidic acid phosphatase protein, putative	-3.7
Tb08.27P2.110	hypothetical protein, conserved, phosphatase protein	-3.6
Tb927.10.10220	PAG2	-3.5
Tb927.5.440	trans-sialidase, putative	-2.8
Tb927.10.10240	PAG1	-2.6
Tb11.55.0028	ESAG3	-2.6
Tb927.8.3620	ESAG10	-2.3
Tb927.6.460	PAG3	-2.1
Tb09.211.4980	LRRP	-1.9
Tb927.7.170	ESAG9	-1.9
Tb11.01.0725	cation transporter	-1.9
Tb927.8.6010	hypothetical predicted multi-pass transmembrane protein	-1.8
Tb927.3.3431	rRNA 5.8S (M3)	-1.8
Tb927.3.3435	rRNA large subunit delta (M2)	-1.6
Tb927.8.2861	SRP RNA, 7SL	-1.6
Tb08.27P2.70	hypothetical protein	-1.6
Tb11.22.0007	hypothetical protein, conserved	-1.6
Tb927.7.190	thimet oligopeptidase A, putative	-1.6
Tb11.53.0001	hypothetical protein	-1.6
Tb927.10.1560	hypothetical protein	-1.5
Tb927.3.3434	rRNA large subunit beta	-1.5
Tb927.5.4170	histone H4, putative	-1.5
Tb927.7.1320	HSP10	-1.5
Tb11.02.5280	glycerol-3-phosphate dehydrogenase	-1.5
Tb927.2.2020	expression site-associated gene 3 (ESAG3) protein	-1.5
Tb11.01.3805	microtubule-associated protein corset-associated protein 1	-1.5
Tb927.3.3432	rRNA large subunit alpha	-1.4
Tb11.01.0730	cation transporter, putative	-1.4
Tb927.3.590	adenosine transporter, putative	-1.4
Tb11.02.0360	hypothetical protein, conserved, phosphatase domain	-1.4
Tb927.10.5840	translation elongation factor 1-beta	-1.4
Tb927.7.4170	fatty acid elongase, putative	-1.4
Tb927.7.4570	inosine-guanine nucleoside hydrolase	-1.4
Tb927.1.3830	glucose-6-phosphate isomerase, glycosomal	-1.3
Tb09.211.0540	fructose-1,6-bisphosphatase	-1.3
Tb927.7.1120	trypanothione/tryparedoxin dependent peroxidase 1	-1.3
Tb11.01.6800	1-acyl-sn-glycerol-3-phosphate acyltransferase, putative	-1.3
Tb09.244.2070	small GTPase, putative	-1.3
Tb11.42.0007	histone H1, putative	-1.3

Tb927.10.14160	aquaporin 3, putative	-1.3
Tb09.160.3710	proliferative cell nuclear antigen (PCNA), putative	-1.3
Tb927.4.4860	amino acid transporter 8, putative	-1.3
Tb927.6.3750	heat shock 70 kDa protein, mitochondrial precursor	-1.2
Tb927.8.740	nucleolar RNA-binding protein	-1.2
Tb927.10.10610	protein tyrosine phosphatase, putative	-1.2
Tb927.10.12330	ZC3H34	-1.2
Tb927.8.7640	AATP1	-1.2
Tb927.1.4830	PLA1 (phospholipase A1)	-1.2
Tb11.55.0011	hypothetical protein, conserved, Emopamil-binding motif	-1.2
Tb927.6.2170	co-chaperone GrpE	-1.2
Tb927.4.2310	asparaginyl-tRNA synthetase, putative	-1.2
Tb927.10.5620	ALD (fructose-bisphosphate aldolase, glycosomal)	-1.2
Tb11.01.0780	hypothetical protein, conserved, TFAR19-motif	-1.2
Tb927.7.2870	histone H2A, putative	-1.2
Tb927.10.8980	hypothetical protein, conserved	-1.2
Tb927.1.2490	histone H3, putative	-1.2
Tb927.3.3270	TbPFK (ATP-dependent phosphofructokinase)	-1.1
Tb11.02.3440	hypothetical protein, conserved	-1.1
Tb927.10.5750	hypothetical protein, conserved	-1.1
Tb927.3.3429	rRNA small subunit	-1.1
Tb927.3.3429	rRNA small subunit	-1.1
Tb927.3.3433	rRNA large subunit gamma (M1)	-1.1
Tb927.10.5120	KREPA6 (MP18)	-1.1
Tb927.10.4990	CRK3 (CDC2-related protein kinase)	-1.1
Tb11.02.0100	carboxypeptidase, putative	-1.1
Tb927.10.10980	heat shock protein 83	-1.1
Tb927.10.14170	Aquaporin 2	-1.1
Tb09.211.1360	hypothetical protein, conserved	-1.1
Tb927.10.14140	PYK1 (pyruvate kinase 1)	-1.1
Tb927.8.6440	RPB20	-1.1
Tb927.10.10930	heat shock protein, putative	-1.1
Tb927.10.6060	DNA-binding protein HEXBP, putative	-1.0
Tb09.211.1690	hypothetical protein, conserved, CS domain	-1.0
Tb927.6.2360	adenosine kinase, putative	-1.0
Tb11.02.4520	amino acid permease/transporter, putative	-1.0
Tb11.18.0006	CAT (carnitine O-acetyltransferase, putative)	-1.0
Tb927.10.3210	delta-1-pyrroline-5-carboxylate dehydrogenase, putative	-1.0
Tb927.7.4470	hypothetical protein, conserved	-1.0
Tb11.01.7600	amino acid transporter, putative	-1.0
Tb09.211.4820	hypothetical protein, conserved, EF-hand motif	-1.0
Tb11.02.5270	haloacid dehalogenase hydrolase, putative	-1.0
Tb927.7.5920	MST (mercaptopyruvate sulfurtransferase, putative)	-1.0
Tb927.10.4500	mRNA capping methyltransferase, putative	-1.0
Tb927.8.5120	cytochrome c	-1.0
Tb927.7.1300	protein disulfide isomerase, putative	-1.0

Tb09.211.4870	RNA 3'-terminal phosphate cyclase-like protein	-1.0
Tb927.10.10450	hypothetical protein	-1.0
Tb927.5.3830	dihydroorotate oxidase	-1.0
Tb927.7.4060	calpain-like cysteine peptidase	-1.0
Tb09.211.4970	hypothetical protein, unlikely	-1.0
Tb927.5.1550	mitochondrial carrier protein	-1.0
Tb927.8.5450	AAT6	-1.0
Tb927.5.4600	hypothetical protein, conserved, Leu-rich domain	-1.0
Tb927.10.3200	U2AF35	-1.0
Tb927.1.2460	hypothetical protein, unlikely	-1.0
Tb09.160.3270	eukaryotic initiation factor 4a, putative	-1.0
Tb927.7.1470	ATPase subunit 9, putative	-1.0
Tb11.02.4700	14-3-3 protein	-1.0
Tb927.5.4140	hypothetical protein, conserved, Zn/Fe permease domain	-1.0
Tb927.4.4390	hypothetical protein	-1.0
Tb927.8.4380	hypothetical protein, conserved	-1.0
Tb927.4.4540	hypothetical protein, conserved, LSD1-like domain	-1.0
Tb927.3.5660	UDP-Gal or UDP-GlcNAc-dependent glycosyltransferase	-1.0
Tb927.8.6760	IgE-dependent histamine-releasing factor, putative	-1.0
Tb11.01.7120	heat shock protein Hsp20, putative	-1.0
Tb927.1.710	PGKB (phosphoglycerate kinase B)	-1.0
Tb927.1.2440	hypothetical protein, unlikely	-1.0
Tb927.1.2500	hypothetical protein, unlikely	-1.0
Tb11.02.3690	hypothetical protein, conserved	-1.0
Tb11.01.2900	protein kinase, putative	-1.0
Tb927.5.4500	TbSAR1 (ras-like small GTPase)	-1.0
Tb927.1.2540	hypothetical protein, unlikely	-1.0
Tb927.7.4770	PPIase	-1.0
Tb11.01.7070	hypothetical protein, conserved	-1.0

Table S2. List of transcripts regulated at least 2-fold after adition of guanosine to late log cultures

Transcripts whose abundance is altered at least ± 2-fold (log2 fold change = ±1.0) upon adition of guanosine to late log procyclic trypanosomes.

Upregulated

Systematic name	Description of gene product	log2 FC
Tb927.10.10230	PAG5	4.5
Tb927.10.10210	PAG4	3.2
Tb927.10.10220	PAG2	3.0
Tb927.10.10240	PAG1	3.0
Tb927.3.3435	rRNA large subunit delta (M2)	3.0
Tb927.8.2861	SRP RNA, 7SL	2.9
Tb927.10.7160	PAG1	2.8
Tb927.3.3432	rRNA large subunit alpha	1.9
Tb927.10.11990	RNA-binding protein	1.7
Tb927.3.3429	rRNA small subunit	1.7
Tb09.211.4870	RNA 3'-terminal phosphate cyclase-like protein	1.7
Tb927.10.10010	60S acidic ribosomal protein, putative	1.7
Tb927.10.6630	HEL64	1.7
Tb927.3.1350	hypothetical protein, conserved, WD40 repeat motif	1.6
Tb927.3.3434	rRNA large subunit beta	1.6
Tb11.02.1670	hypothetical protein, conserved, peptidase M24 motif, DNA-binding	1.5
Tb927.8.4450	RBP11	1.5
Tb927.10.12330	ZC3H34	1.5
Tb927.10.12430	hypothetical protein, conserved, nucleolar complex 2 motif	1.5
Tb09.211.2230	hypothetical protein, conserved, TPR-like motif	1.5
Tb927.4.2310	asparaginyl-tRNA synthetase, putative	1.5
Tb11.01.6600	PUF7	1.5
Tb927.7.270	ribosome biogenesis protein, putative	1.4
Tb927.3.2750	hypothetical protein, conserved	1.4
Tb09.244.2790	rRNA processing protein, putative	1.4
Tb11.02.0570	hypothetical protein, conserved, WD40 repeat motif	1.4
Tb927.10.1560	hypothetical protein, conserved	1.4
Tb927.10.2310	hypothetical protein, conserved	1.4
Tb927.10.9200	hypothetical protein, conserved, NERD motif	1.3
Tb11.02.0100	carboxypeptidase, putative	1.3
Tb927.8.2000	NCP1 (peptidyl-prolyl cis-trans isomerase)	1.3
Tb927.2.1443	5.8S(M3) ribosomal RNA	1.3
Tb927.7.190	thimet oligopeptidase A, putative	1.3
Tb09.211.0180	hypothetical protein, conserved, pescadillo motif	1.3
Tb927.8.5490	hypothetical protein, conserved, nucleolar Nop52 motif	1.3
Tb927.4.2630	ATP-dependent DEAD/H RNA helicase, putative	1.3
Tb927.8.1270	hypothetical protein, conserved	1.3
Tb927.8.2600	hypothetical protein, conserved, WD40 repeat motif	1.3
Tb09.160.1560	hypothetical protein, conserved, armadillo motif	1.2
Tb11.18.0014	hypothetical protein, conserved, CBF motif	1.2
Tb927.10.14870	hypothetical protein, conserved	1.2
Tb927.10.3560	arginine N-methyltransferase, putative	1.2
Tb927.5.3810	OMPDCase-OPRTase, putative	1.2
Tb11.01.0820	hypothetical protein, conserved, P-loop motif	1.2
Tb11.02.0620	NOG1	1.2
Tb11.01.7680	DRBD10	1.2
Tb927.3.3670	TRRM3	1.2
Tb09.160.0380	hypothetical protein, conserved, DPH-type Zn finger motif	1.2

Tb927.2.4550	FtsJ cell division protein, putative	1.2
Tb927.1.880	hypothetical protein, conserved, midasin motif, ATPase motif	1.1
Tb927.4.3840	nucleolar protein, putative	1.1
Tb927.6.2780	U3 small nuclear ribonucleoprotein (snRNP), putative	1.1
Tb927.8.5990	hypothetical protein, conserved, WD40 repeat motif	1.1
Tb11.02.2920	hypothetical protein, conserved	1.1
Tb11.02.0240	TbNST	1.1
Tb927.8.5040	hypothetical protein, conserved, NEP1 motif	1.1
Tb11.02.4570	PUF10	1.1
Tb927.10.13310	RPB5z	1.1
Tb11.02.0110	hypothetical protein, conserved, armadillo motif	1.1
Tb927.8.4820	eukaryotic translation initation factor 4 gamma	1.1
Tb09.211.1800	hypothetical protein, conserved, LisH dimerisation motif	1.1
Tb927.1.1700	hypothetical protein, conserved, AATF motif	1.1
Tb11.01.2250	hypothetical protein, conserved, WD40 repeat motif	1.1
Tb927.10.5670	NAT1 (N-acetyltransferase subunit)	1.1
Tb927.10.6320	hypothetical protein, conserved, CBF motif, nucleolar complex protein	1.1
Tb927.5.840	hypothetical protein, conserved, KRL1 motif	1.1
Tb927.7.4220	hypothetical protein, conserved, WD40 repeat motif	1.1
Tb927.10.6060	DNA-binding protein HEXBP, putative	1.0
Tb927.10.8310	acetyltransferase, putative	1.0
Tb927.5.1150	RNA helicase Prp43	1.0
Tb927.10.1720	ATP-dependent DEAD/H RNA helicase, putative	1.0
Tb927.7.970	hypothetical protein, conserved, NMD3 motif	1.0
Tb09.160.4830	hypothetical protein, conserved	1.0
Tb11.02.5210	RNA binding protein, putative	1.0
Tb927.7.4970	glutamine synthetase, putative	1.0
Tb927.6.2170	co-chaperone GrpE	1.0
Tb927.3.2830	hypothetical protein, conserved, BRIX motif	1.0
Tb927.8.5090	RPA190	1.0
Tb927.10.9780	ATP-dependent DEAD/H RNA helicase, putative	1.0
Tb927.5.1560	ATP-dependent DEAD/H RNA helicase, putative	1.0
Tb927.3.2470	PUF8	1.0
Tb927.10.15170	hypothetical protein, conserved, RNA helicase DDX18 motif	1.0
Tb11.01.3100	hypothetical protein, conserved	1.0
Tb927.8.6440	RPB20	1.0
Tb11.01.0950	hypothetical protein, conserved, nucleolar NOL1 motif	1.0
Tb11.18.0006	CAT (carnitine O-acetyltransferase, putative)	1.0
Tb09.211.2960	hypothetical protein, conserved, KH motif	1.0
Tb927.5.4420	nucleolar RNA helicase Gu, putative	1.0
Tb927.10.13270	predicted WD40 repeat protein	1.0
Tb927.5.4100	hypothetical protein, conserved, WD40 repeat motif	1.0
Tb927.8.1410	hypothetical protein, conserved,ribosome biogenesis protein, TSR1	1.0
Tb927.6.2650	hypothetical protein, conserved, Initiation factor eIF-4 gamma	1.0
Tb927.3.3510	hypothetical protein, conserved	1.0
Tb927.4.4860	amino acid transporter 8, putative	1.0

Downregulated

Tb11.02.1105	NT8	-2.4
Tb927.3.4500	cFUM, cytosolic fumarase	-2.2
Tb11.01.7190	NADH-cytochrome b5 reductase	-2.0
Tb927.10.12270	hypothetical protein, conserved (SAM methyltransferase motif)	-1.7
Tb927.10.2560	mMDH, mitochondrial malate dehydrogenase	-1.7
Tb11.01.4560	variant surface glycoprotein (VSG)-related, putative	-1.6
Tb927.7.6220	protein kinase, putative	-1.4
Tb927.5.1360	NDRT, nucleoside 2-deoxyribosyltransferase	-1.4
Tb927.10.2010	hexokinase	-1.4
Tb927.1.4440	hypothetical protein, conserved	-1.4
Tb927.10.9340	hypothetical protein	-1.4
Tb09.211.0350	adenylate kinase	-1.3
Tb927.7.4390	threonine synthase, putative	-1.3
Tb11.01.7880	CAP17 (microtubule-associated protein corset-associated protein 17)	-1.3
Tb927.1.710	PGK-B (phosphoglycerate kinase B)	-1.3
Tb927.10.13390	hypothetical protein, conserved	-1.2
Tb09.160.4320	hypothetical protein, unlikely	-1.2
Tb927.10.13530	hypothetical protein	-1.2
Tb09.v1.0380	SpSyn (spermidine synthase)	-1.2
Tb927.8.3530	glycerol-3-phosphate dehydrogenase [NAD+], glycosomal	-1.2
Tb927.2.2770	hypothetical protein, conserved	-1.2
Tb927.10.13690	hypothetical protein, conserved	-1.2
Tb927.2.5980	HSP104	-1.2
Tb927.7.1930	nucleoside diphosphatase, putative	-1.2
Tb927.8.5460	44 kDa calflagin	-1.2
Tb09.211.3560	glk1 (glycerol kinase, glycosomal)	-1.1
Tb927.8.5440	24 kDa calflagin	-1.1
Tb927.10.11970	kynurenine aminotransferase, putative	-1.1
Tb927.8.5470	17 kDa calflagin	-1.1
Tb927.8.980	phosphoacetylglucosamine mutase, putative	-1.1
Tb09.v1.0230	hypothetical protein, unlikely	-1.1
Tb927.8.5465	24 kDa calflagin	-1.1
Tb927.10.14000	ACO (aconitase)	-1.1
Tb927.6.4020	hypothetical protein, conserved	-1.1
Tb09.211.3060	hypothetical protein, unlikely	-1.1
Tb927.10.3040	hypothetical protein, conserved	-1.0
Tb09.160.4380	succinate dehydrogenase, putative	-1.0
Tb927.8.4430	uridine phosphorylase	-1.0
Tb927.8.4770	small GTP-binding protein Rab18	-1.0
Tb927.7.310	hypothetical protein, conserved, Glutathione S-transferase mnotif	-1.0
Tb927.4.1360	hypothetical protein, conserved, Glycoside hydrolase-type	-1.0
Tb927.1.1580	cytochrome c oxidase assembly factor, putative	-1.0
Tb927.8.4630	hypothetical protein, conserved	-1.0

A

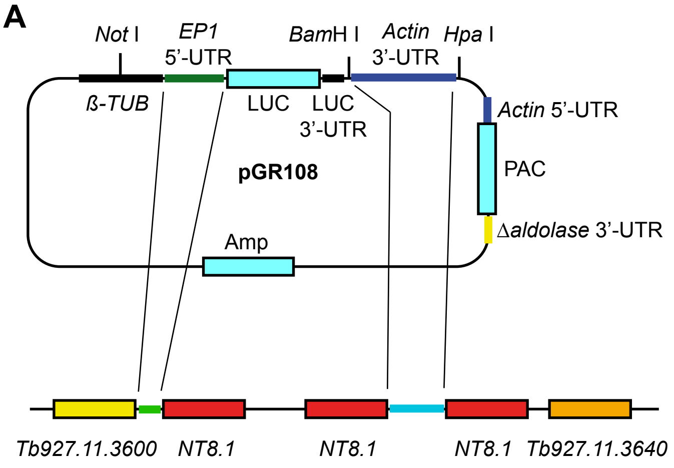

B

**Plasmid**	**Expression cassette**
pGR100	EP1-LUC-mMDH
pGR108	EP1-LUC-ACT
pGR197	NT8-LUC-ACT
pGR281	EP1-LUC-NT8
pGR282	NT8-LUC-NT8
pGR284	EP1-LUC-NT8Δ1
pGR285	EP1-LUC-NT8Δ2
pGR286	EP1-LUC-NT8Δ3
pGR287	EP1-LUC-NT8Δ4
pGR290	EP1-LUC-NT8Δstem-loop
pGR291	EP1-LUC-mMDH + stem-loop
pGR292	EP1-LUC-ACT + stem-loop
pGR293	EP1-LUC-ACT + short stem-loop
pGR295	EP1-LUC-ACT + short stem-loop, unstructured
pGR299	EP1-LUC-ACT + short stem-loop, 3-nt loop
pGR300	EP1-LUC-ACT + short stem-loop, stem with non structurally synonymous mutations
pGR300	EP1-LUC-ACT + short stem-loop, stem with structurally synonymous mutations

Supplementary Figure S1. Plasmids used in this work. (**A**) All constructs are based in plasmid pGR108 (Estevez, 2008) that expresses a luciferase reporter gene under the control of procyclin EP1 5'-UTR and actin 3'-UTR. (**B**) List of expression vectors.

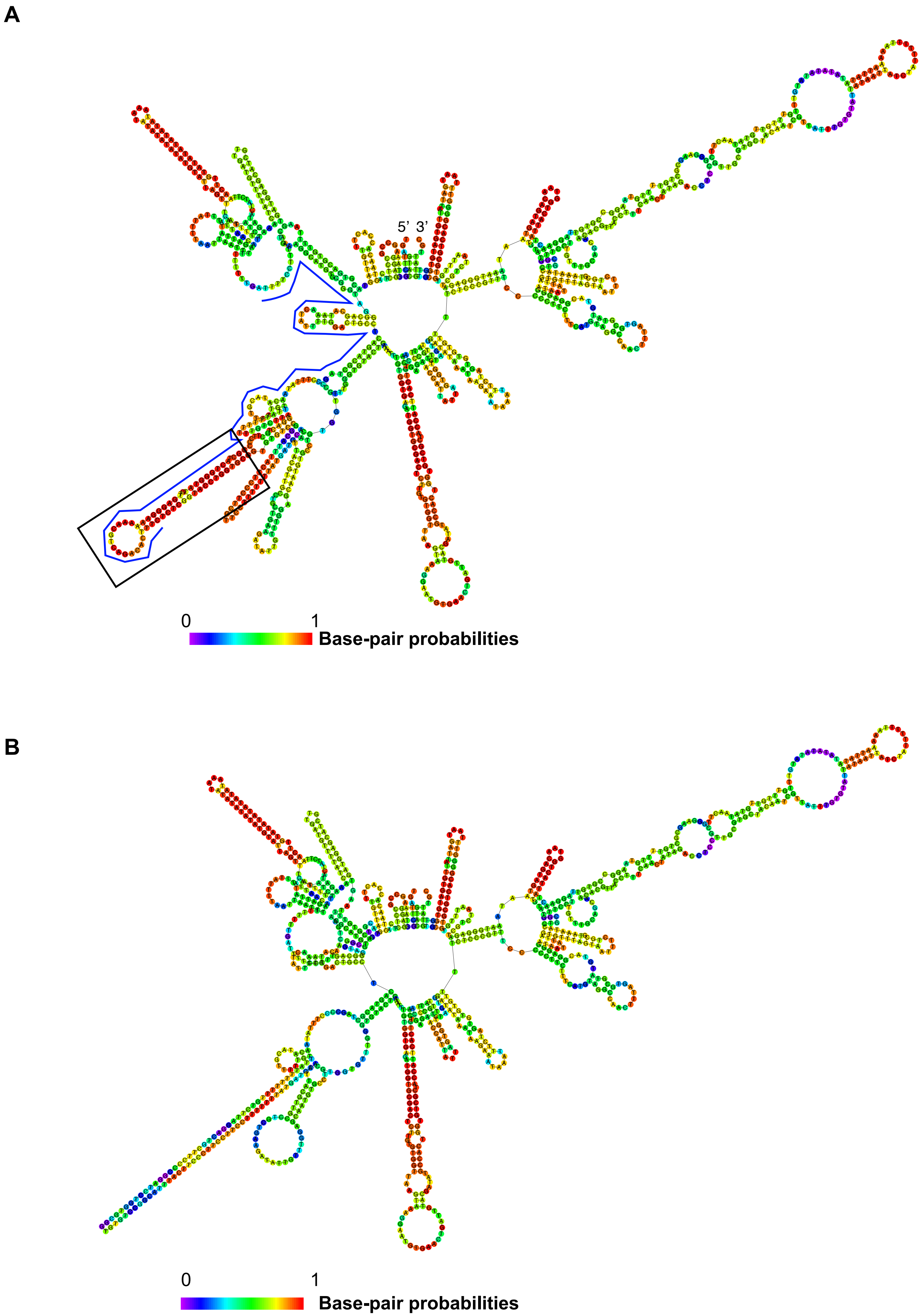

Supplementary Figure S2. Predicted secondary structure of the 3’-UTR of NT8 containing (A) or lacking (B) the regulatory stem-loop. The region corresponding to deletion Δ2 is indicated with a blue line. The regulatory stem-loop is shown boxed.

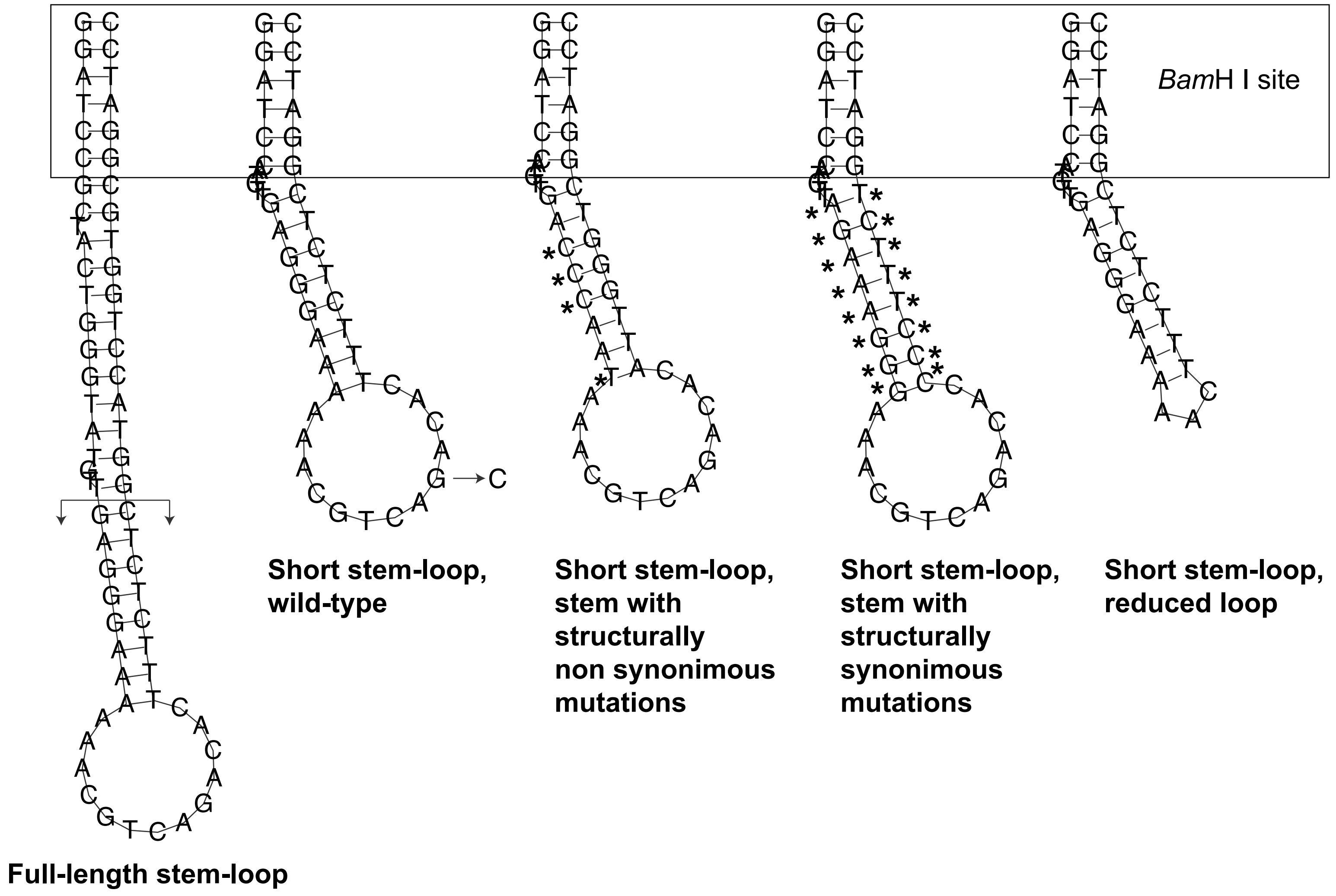

Supplementary Figure S3. Sequence and predicted structures of the mutant versions of the regulatory stem-loop used in this work. The *Bam*H I site used for cloning is shown boxed. A shorter version of the full-length stem-loop, corresponding to the sequence below the arrows in the full-length version, gave a similar effect on luciferase expression, and was used to generate the different mutant versions. A G to C point mutation in the loop is predicted to disrupt the whole structure (see Figure S4). Mutations are indicated by asterisks.

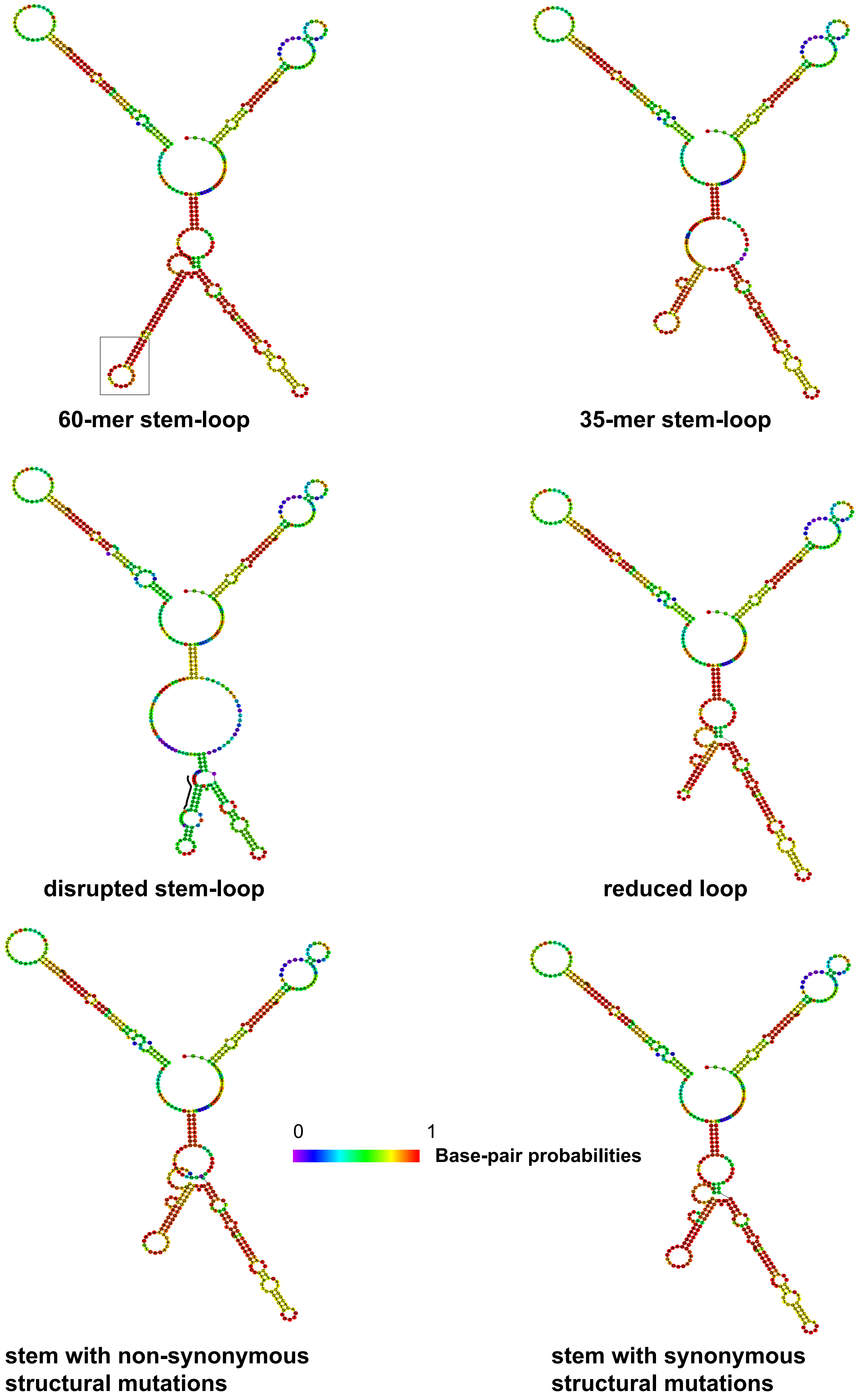

Supplementary Figure S4. Predicted secondary structures of the 3'-UTR of LUC-ACT reporter mRNAs containing different versions of the regulatory stem-loop. The regulatory stem-loop is shown boxed. In the disrupted stem-loop structure, the sequence corresponding to the loop is indicated with a line.

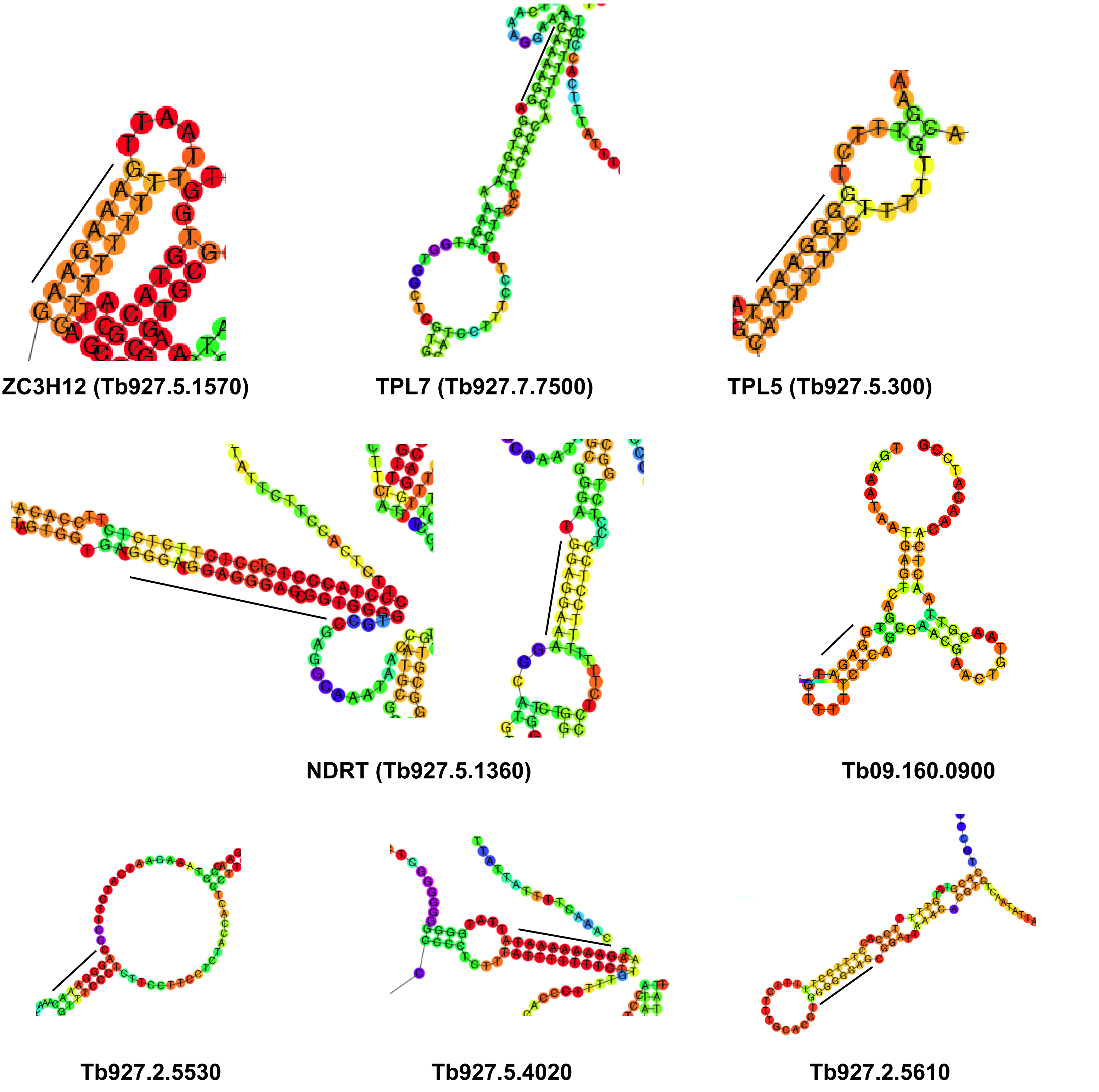

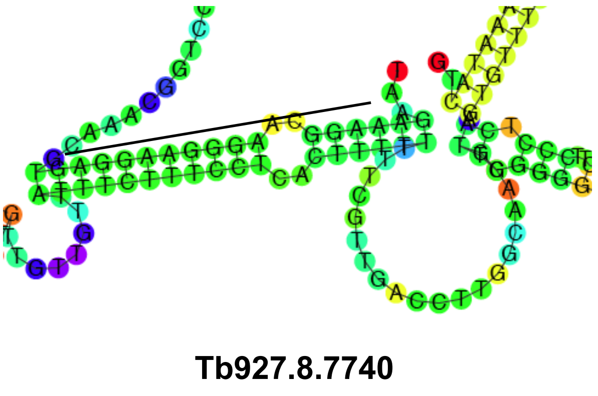

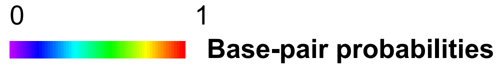

Supplementary Figure S5. Regions in the 3'-UTR of the most upregulated mRNAs during late log phase which are similar to the *NT8* regulatory stem-loop. Purine-pyrimidine double-stranded regions are indicated with a line.
